# George N. Papanicolaou (1883–1962): The Pioneer of Cytology and Early Cancer Detection

**DOI:** 10.7759/cureus.68999

**Published:** 2024-09-09

**Authors:** Christos S Avdulla, Ntaniela Tachirai

**Affiliations:** 1 Department of Public Health, University of Patras, Patras, GRC; 2 Department of Public Health Policy, University of West Attica, Athens, GRC

**Keywords:** cancer detection, cytology, george papanicolaou, historical vignette, pap test, public health

## Abstract

George N. Papanicolaou (1883-1962) is recognized as a pioneer in cytology and early cancer detection, whose groundbreaking work revolutionized women’s healthcare. His development of the Pap smear, a simple yet powerful screening tool, dramatically improved the early diagnosis of cervical cancer, leading to a significant reduction in mortality rates and saving millions of lives worldwide. Born in Greece and extensively trained in medicine and biology, Papanicolaou conducted meticulous research at Cornell University, laying the foundation for modern preventive medicine. This historical vignette explores Papanicolaou’s life, transformative achievements, and the innovative processes he championed. By examining his enduring contributions, we highlight his profound impact on public health and medical practice, which continues to inspire and guide healthcare professionals across generations.

## Introduction and background

**Figure 1:**
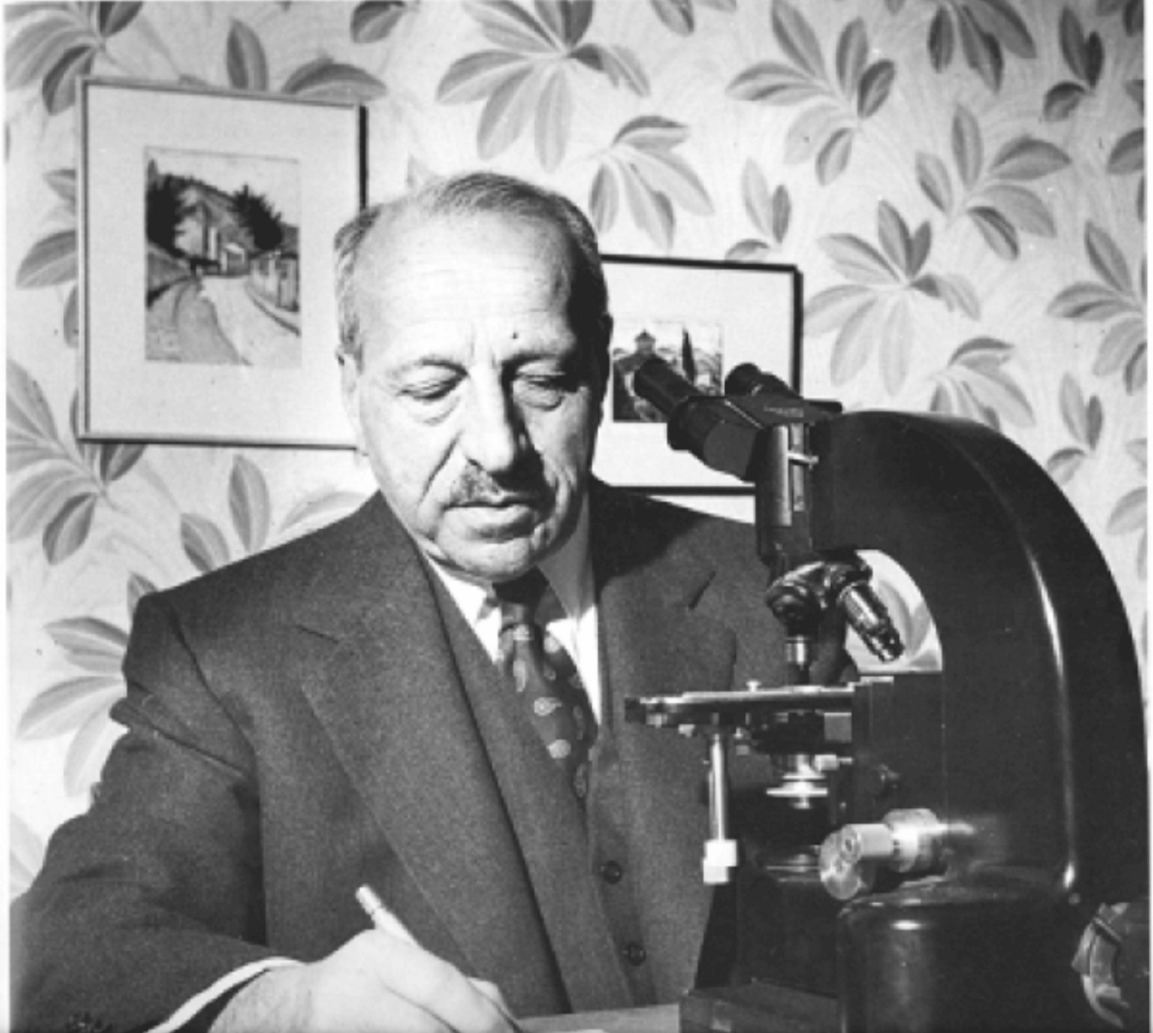
George N. Papanicolaou (1883–1962) Source: Institute of Documentation, Information, and Research on Cancer Georgios N. Papanikolaou.  Public domain [3].

George N. Papanikolaou (George Nicholas Papanicolaou or Georgios Nikolaou Papanikolaou) is synonymous with cytology and early cancer detection, particularly cervical cancer (Figure 1). Born on May 13, 1883, in the small town of Kymi, Greece, Papanicolaou grew up in a family that deeply valued education and intellectual pursuits. His father, a respected physician, served as an early role model and inspired his initial interest in medicine, leading him to study at the University of Athens, where he graduated in 1904 [1,2].

After earning his medical degree, Papanicolaou continued his studies in Germany, enrolling in biology and zoology programs at the Universities of Munich and Jena. This formative period exposed him to cutting-edge research and provided a strong foundation in experimental research methods. During this time, he developed a keen interest in the microscopic study of cells, which later became crucial to his pioneering work in cytology [4,5]. In 1913, Papanicolaou emigrated to the United States and joined Cornell University Medical College in New York City. It was here that he began the research that would eventually lead to the development of the Pap smear [6-8]. 

This article aims to provide a comprehensive examination of the life of George N. Papanicolaou. It highlights his pioneering achievements and the innovative processes he developed, while also inspiring current and future healthcare professionals to build upon his legacy in early diagnosis and preventive care.

## Review

Contributions to cytology and cancer detection

Papanicolaou's most significant contribution to medicine is the development of the Pap smear, a revolutionary test that enables the early detection of cervical cancer through the microscopic examination of exfoliated cells from the cervix. This breakthrough was pivotal not only for women's health but also marked the advent of cytology as a crucial diagnostic tool in medicine [8,9].

His journey toward developing the Pap smear began with early research on the reproductive system of guinea pigs. Observing cellular changes in vaginal smears that correlated with the estrous cycle stages, he hypothesized that similar changes in human smears could identify abnormal, precancerous, or cancerous conditions. His pioneering idea was to use cytological examination to detect cancer at an early, more treatable stage [9,10].

In 1928, Papanicolaou presented his preliminary findings, suggesting that cancerous cells could be identified in vaginal smears. Despite initial skepticism from the medical community, he continued refining his techniques and gathering more data. It wasn't until the early 1940s, after years of painstaking research and collaboration with gynecologist Herbert Traut, that the Pap smear was validated through clinical trials. Their joint 1943 publication, Diagnosis of Uterine Cancer by the Vaginal Smear, provided compelling evidence for the Pap test's efficacy, leading to its widespread adoption [10,11].

The introduction of the Pap smear had an immediate and profound impact on public health. Before its widespread use, cervical cancer was one of the leading causes of death among women. The ability to detect abnormal cells before they progressed to invasive cancer allowed for early intervention, drastically reducing cervical cancer mortality. Over the following decades, the Pap smear became the cornerstone of cervical cancer screening programs worldwide, contributing to a dramatic decline in the disease's incidence and mortality rates [12,13].

Despite the challenges he faced, including limited funding and initial resistance from the medical community, Papanicolaou's perseverance and commitment to his research never wavered. His work exemplifies the power of scientific inquiry and the importance of innovation in advancing medical practice. By the time of his death in 1962, the Pap smear had become a standard procedure in gynecological care, and Papanicolaou himself had been recognized globally as a pioneer in the fight against cancer [12-14].

Innovative processes and methods

Papanicolaou's work was characterized by a relentless pursuit of precision and innovation. His approach to cytology was methodical yet groundbreaking, developing techniques far ahead of their time and establishing cytology as a critical diagnostic medicine field [9].

One of his key innovations was developing a reliable staining method for exfoliated cells, which allowed for the clear differentiation between normal and abnormal cellular structures. The Papanicolaou stain, which he perfected, enabled the visualization of cell morphology with unprecedented clarity. This staining technique became a cornerstone of cytological analysis, allowing for the accurate identification of precancerous and cancerous cells in the cervix and other tissues [9,15].

Papanicolaou's meticulous process involved collecting cells from the cervix using a simple scraping method, followed by applying his staining technique. This process was non-invasive and could be performed easily in a clinical setting, making it an accessible and practical tool for routine screening. His innovation lay not just in the procedure itself but in the combination of technique, staining, and systematic analysis, providing a reliable and reproducible method for early cancer detection [11].

Moreover, Papanicolaou's insistence on correlating cytological findings with clinical outcomes was crucial in validating the Pap smear's effectiveness. He conducted extensive longitudinal studies, often following patients over many years to observe the progression from normal cells to cancerous ones. This rigorous research approach provided the statistical evidence needed to convince the medical community of the test's value, leading to its eventual widespread adoption [12,13].

Papanicolaou's methods were not only innovative but also comprehensive. He explored the diagnostic potential of cytology beyond cervical cancer, applying his techniques to other types of cancer and diseases. His work laid the foundation for exfoliative cytology, which today is used to detect a wide range of conditions, including lung, bladder, and gastrointestinal cancers. Papanicolaou's emphasis on early disease detection through non-invasive means has had a lasting impact on preventive medicine and public health [12,15,16].

His interdisciplinary approach was also a significant factor in his success. Papanicolaou collaborated with clinicians, pathologists, and public health officials to refine his techniques and ensure their practical application in the medical field. This collaboration was essential in transitioning the Pap smear from a research tool to a standard practice in women's healthcare [17].

Papanicolaou's dedication to innovation extended beyond the laboratory. He was also a strong advocate for public health, emphasizing the importance of early detection and preventive care. His work demonstrated that technological innovation in medicine must be accompanied by a commitment to patient care and public health, principles that continue to guide medical practice [14].

Enduring impact and legacy

Papanicolaou's contributions to medicine have left an enduring legacy that continues to shape the landscape of public health and cancer prevention. The development of the Pap smear revolutionized the early detection of cervical cancer, transforming it from one of the leading causes of death among women into a largely preventable disease. The test's introduction in the 20th century led to a dramatic decline in cervical cancer mortality rates worldwide, demonstrating the profound impact of Papanicolaou's work on global health [13,17,18].

The Pap smear's success established cytology as a critical diagnostic tool, leading to the expansion of the field beyond cervical cancer. This expansion of cytology has saved countless lives by enabling the early diagnosis and treatment of various cancers, further solidifying Papanicolaou's legacy as a pioneer in medical science [18].

Papanicolaou's work also highlighted the importance of preventive medicine, a concept now central to modern healthcare. His emphasis on early detection to prevent disease progression has influenced public health strategies globally. Cervical cancer screening programs, now standard practice in many countries, owe their existence to the principles established by Papanicolaou. These programs have become a model for other cancer screening initiatives, demonstrating the broader applicability of his approach to preventive care [12,19].

Beyond his technical innovations, Papanicolaou's dedication to research, patient care, and public health advocacy has inspired generations of healthcare professionals and researchers. His work ethic, perseverance in the face of challenges, and commitment to improving patient outcomes continue to serve as a model for aspiring medical scientists. Papanicolaou's life and work remind us of the power of scientific inquiry and the profound impact a single individual can have on global health [9,14-18].

## Conclusions

George N. Papanicolaou's pioneering work in cytology and cancer detection represents one of the most significant advancements in 20th-century medicine. His development of the Pap smear transformed women's healthcare, providing a reliable method for the early detection of cervical cancer that continues to save lives to this day. Papanicolaou's meticulous research, innovative techniques, and commitment to patient care laid the foundation for modern cytopathology and underscored the critical importance of early disease detection in improving public health. His legacy continues to inspire healthcare professionals and researchers around the world, highlighting the enduring impact of his contributions to medical science.
